# Using hyperhomocysteinemia and body composition to predict the risk of non-alcoholic fatty liver disease in healthcare workers

**DOI:** 10.3389/fendo.2022.1063860

**Published:** 2023-01-06

**Authors:** Xiaoyan Hao, Honghai He, Liyuan Tao, Peng Wang

**Affiliations:** Medical examination center, Peking University, Third Hospital, Beijing, China

**Keywords:** homocysteine, insulin resistance, neck-circumference, abdominal visceral fat area, skeletal-muscle content

## Abstract

**Purpose:**

This study investigated associations between serum homocysteine levels, body composition, and the probability of having nonalcoholic fatty liver disease (NAFLD) in Chinese healthcare workers.

**Patients and Methods:**

A total of 4028 healthcare workers were enrolled in this study, and all underwent a physical examination. Body composition was measured using multifrequency bioelectrical impedance analysis.

**Results:**

There were 1507 NAFLD patients (72.26% male, 27.74% female) and 2521 controls (39.83% male, 60.17% female). Body mass index (BMI), waistline, neck-circumference (NC), abdominal visceral fat area (AVFA), total cholesterol (TC), triglycerides (TG), high-density lipoprotein cholesterol (HDL-C), glucose (Glu), homocysteinemia (hcy) were higher in the NAFLD group than controls. Additionally, the skeletal-muscle was associated with a lower risk of NAFLD, whereas BMI, waistline, NC, hyperhomocysteinemia (HHcy) were associated with a higher risk of NAFLD. The best NC cut-off point for NAFLD was 34.45 cm (sensitivity 83.3% and specificity 83.9%) in women with HHcy, and the best skeletal-muscle content cut-off point for NAFLD was 41.335% (sensitivity 74.2% and specificity 65.6%) in men with HHcy.

**Conclusion:**

Interactions between skeletal-muscle content, NC, and HHcy may affect the incidence of NAFLD in healthcare workers. This may provide a novel approach for diagnosing NAFLD.

## 1 Introduction

The prevalence of NAFLD is approximately 25%, and continues to rise along with global obesity rates (1, 2). NALFD, a major pathogenic promoter of steatohepatitis, fibrosis, and hepatocellular carcinoma (3), is thought to be a manifestation of metabolic syndrome in the liver (4). NAFLD has reached epidemic proportions in China, with a prevalence of 30% (5). Risk factors for the development of NAFLD may include advanced age, male gender, obesity, decreased muscle mass and increased visceral fat mass (6, 7). The prevalence of HHcy is 34.61% in northern China (8). Several studies have reported that serum hcy levels were higher in patients NAFLD (9, 10), and other literature revealed that NAFLD patients were associated with an increased risk of HHcy (11). HHcy maybe a cause of the hepatic steatosis of NAFLD (12). Insulin resistance is associated with both HHcy (13, 14) and NAFLD (15), and may be one of the pathologic mechanisms that underly the relationship between NAFLD and HHcy. Body composition metrics include NC, skeletal muscle content, AVFA, and abdominal circumference (16, 17). NC and AVFA are predictors of NAFLD (18). Decreased skeletal muscle mass and increased AVFA are thought to increase the risk of NAFLD (19). There is a robust association between HHcy and low skeletal muscle mass (20). A study shows total body fat proportion were significantly associated with increases in Hcy (21). However, there are few studies that have demonstrated the NAFLD is caused by body composition combined with HHcy. This study sought to clarify associations between HHcy, body composition, and the probability of having NAFLD in healthcare workers.

## 2 Materials and methods

### 2.1 Study design and subject

This study was a single-center,retrospective, case-controlled study. Data were collected from January 2021 to December 2021 at the Medical Examination Center of Peking University Third Hospital. This study was conducted in accordance with the Declaration of Helsinki. All healthcare workers provided written informed consent prior to participation in the study. Protocols involving human participants were reviewed and approved by the Institutional Ethics Committee of Peking University Third Hospital(project:M2021661). All subjects worked in the Peking University Third Hospital and underwent liver ultrasonography that was performed using the same equipment by the same experienced radiologist. NAFLD was diagnosed according to relevant guidelines and regulations (22). Patients were excluded if they had the following conditions: viral hepatitis; drug-induced hepatitis; excessive alcohol consumption; primary biliary cirrhosis; or severe liver, kidney, or thyroid dysfunction (23). Normal control individuals were selected based on abdominal ultrasonography, but those with liver disease were excluded. A total of 4,065 adults participated in this study, and all underwent both a physical examination and a body composition analysis. Among these, 37 patients were excluded for the following reasons: 9 did not sign an informed consent form, 10 did not provide a completed questionnaire, and 18 participants had inadequate blood samples. A total of 4,028 healthcare workers were ultimately included in this study.

### 2.2 Data collection, body fat and muscle mass analysis

A physical examination, history, and body composition measurements were performed by a single trained health care provider. Subject histories included family history, drug history, smoking status, and alcohol intake. Abdominal ultrasound (HI VISION Ascendu, Japan) examinations were routinely performed in a health check-up at our medical examination center. Body composition measurements was obtained *via* bioelectrical impedance analysis using InBody770 (Biospace Co.,Lid, Korea) (24). NC, waistline, AVFA, and skeletal muscle content measurements were performed (25).

### 2.3 Measurement of clinical parameters

Body weight (kg), height (m) and waist circumference (cm) were measured in the standing position. Hcy levels were measured *via* fluorescence detection (F-1080, Hitachi Ltd., Tokyo, Japan) on high-performance liquid chromatography (HPLC; LC-9A, Shimadzu Corp., Kyoto, Japan). Glu was measured using the hexokinase method. TC, HDL-C, TG, alanine aminotransferase (ALT), aspartate transaminase (AST), and hemoglobin (Hb) were using an autoanalyzer (Cobas c 501 autoanalyzer, Roche Diagnostics, Germany).

### 2.4 Statistical analysis

Student’s t-test, one way-ANOVA, chi-squared tests, Fisher’s exact test, Mann-Whitney U test, and binary logistic regression were performed using SPSS version 26.0 (IBM, Armonk, NY, USA). We used receiver operating curve (ROC) analysis to calculate area under the ROC and body composition cutoff values (female and male). P-values < 0.05 were considered statistically significant.

## 3 Results

### 3.1 Clinical characteristics

**Figure 1:**
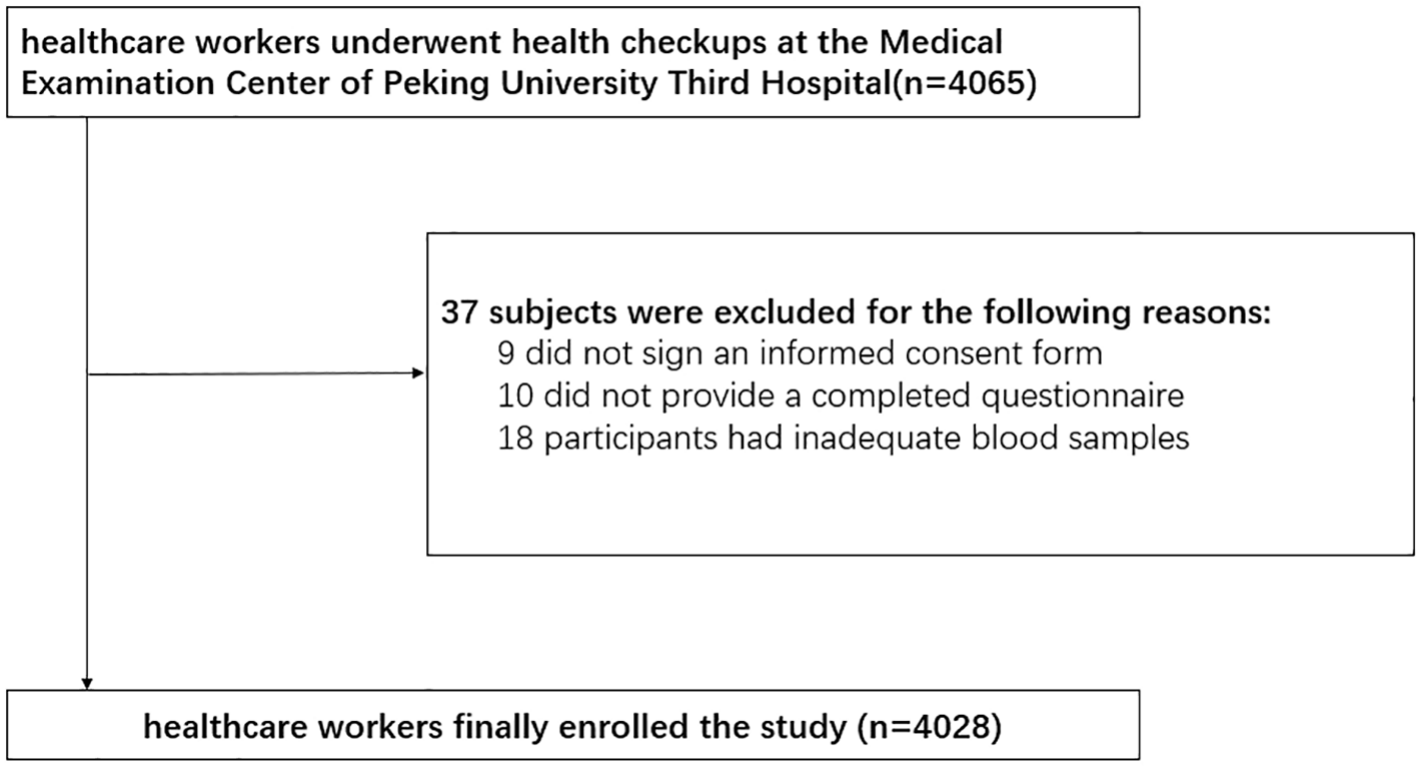
Flowchart for selection of study participants.

A total of 4028 individuals (2093 male, 1935 female) participated in this study (**Figure 1**), including 1507 NAFLD patients (72.26% male, 27.74% female) and 2521 controls (39.83% male, 60.17% female). The average ages of the NAFLD patients and controls were 47.07 ± 9.94 and 43.74 ± 10.12 years, respectively. There were significant differences in age, sex, BMI, waistline, NC, AVFA, skeletal-muscle content, TC, TG, HDL-C, Glu, and Hcy between the NAFLD and control groups (**Table 1**). BMI, waistline, NC, AVFA, TC, TG, HDL-C, Glu, Hcy were significantly higher in the NAFLD group compared with controls. Importantly, the average Hcy level 12.6 in the NAFLD group and 11.1 in controls. Skeletal-muscle content was significantly lower in the NAFLD group compared with controls.

**Table 1:** Baseline clinical characteristics of the study subjects.

Characteristics	NAFLD (1507)	Control (2521)	P-value
Age(year)	47.07 ± 9.94	43.74 ± 10.12	<0.001
male(%)	1089(72.26)	1004(39.83)	<0.001
female(%)	418(27.74)	1517(60.17)	<0.001
BMI(kg/m^2^)	26.97 ± 3.13	22.93 ± 2.77	<0.001
Waistline(cm)	94.81 ± 9.38	82.33 ± 8.04	<0.001
NC(cm)	37.61 ± 2.50	34.21 ± 2.89	<0.001
AVFA(cm^2^)	111.62 ± 34.39	80.53 ± 28.10	<0.001
skeletal-muscle(Kg)	38.23 ± 3.70	39.22 ± 3.99	<0.001
TC(mmol/L)	5.21 ± 0.98	4.99 ± 0.91	<0.001
TG(mmol/L)	1.72(1.27,2.42)	0.97(0.73,1.31)	<0.001
HDL-C(mmol/L)	1.15 ± 0.24	1.41 ± 0.33	<0.001
Glu(mmol/L)	5.81 ± 1.49	5.21 ± 0.84	<0.001
Homocysteine(umol/L)	12.6(10.4,15.7)	11.1(9.2,13.7)	<0.001

BMI, body mass index; skeletal-muscle means skeletal-muscle content; NC, neck circumference; AVFA, abdominal visceral fat area; TC, total cholesterol; TG, triglycerides; HDL-C, high-density lipoprotein cholesterol; Glu, glucose.

### 3.2 Clinical Indicators of HHcy

As shown in **Table 2**, the prevalence of HHcy was 29.13% in the NAFLD group and 17.89% in the control group. In the HHcy group, NC was 38.63 ± 2.14 in the NAFLD group vs 35.95 ± 2.64 in controls while skeletal-muscle content was 39.23 ± 3.31 in the NAFLD group compared with 41.27 ± 3.84 in controls. In normal-Hcy group, NC was 37.19 ± 2.52 in the NAFLD group compared with controls while skeletal-muscle content was 37.83 ± 3.78 in the NAFLD group compared with 38.78 ± 3.88 in controls. Patients with NAFLD had higher waistline, NC and AVFA measurements in the HHcy group than the normal-Hcy group, while skeletal-muscle content was lower in the HHcy group than the normal-Hcy group.

**Table 2:** Clinical characteristics of the HHcy and Normal-Hcy groups.

	HHcy (hcy>15 umol/L)			Normal-Hcy(hcy<15 umol/L)		
	NAFLD(439)	Control(451)	P-value	NAFLD(1068)	Control(2070)	P-value
ALT(U/L)	28(20,40)	18(13,23)	<0.001	25(18,36)	15(11,21)	<0.001
AST(U/L)	24(20,29)	21(18,24)	<0.001	23(19,28)	20(17,23)	<0.001
TC(mmol/L)	5.17 ± 0.94	4.92 ± 0.93	<0.001	5.23 ± 1.00	5.01 ± 0.90	<0.001
TG(mmol/L)	1.82(1.33,2.46)	1.08(0.79,1.47)	<0.001	1.68(1.23,2.38)	0.94(0.72,1.27)	<0.001
HDL-C(mmol/L)	1.09 ± 0.25	1.28 ± 0.30	<0.001	1.17 ± 0.24	1.44 ± 0.33	<0.001
Hemoglobin(g/L)	157.36 ± 11.56	150.90 ± 14.29	<0.001	149.40 ± 15.57	138.92 ± 15.35	<0.001
Glu(mmol/L)	5.68 ± 1.29	5.23 ± 0.83	<0.001	5.87 ± 1.56	5.21 ± 0.84	<0.001
NC(cm)	38.63 ± 2.14	35.95 ± 2.64	<0.001	37.19 ± 2.52	33.84 ± 2.80	<0.001
Waistline(cm)	97.36 ± 9.63	85.16 ± 8.47	<0.001	93.76 ± 9.07	81.71 ± 7.81	<0.001
AVFA(cm^2^)	111.76 ± 35.70	77.83 ± 27.80	<0.001	111.56 ± 33.86	81.11 ± 28.13	<0.001
Skeletal-muscle(Kg)	39.23 ± 3.31	41.27 ± 3.84	<0.001	37.83 ± 3.78	38.78 ± 3.88	<0.001

NC, neck circumference; AVFA, abdominal visceral fat area; TC, total cholesterol; TG, triglycerides; HDL-C, high-density lipoprotein cholesterol; Glu, glucose; ALT, alanine aminotransferase; AST, aspartate transaminase.

### 3.3 Clinical indicators in males and females

As shown in **Table 3**, hcy was 13.7 in the male-NAFLD group and 13.7 in the male-control group. There was no statistical significance between the two groups. Similarly, Hcy was 9.85 in the female-NAFLD group and 9.8 in the female-control group, which was also not statistically significant. AVFA was 106.34 ± 32.47 in the male-NAFLD group compared with 74.38 ± 24.57 in the male-control group. There were significant differences in waistline, NC, AVFA, skeletal-muscle content, TC, TG, HDL-C, Glu, Hb, alanine aminotransferase (ALT), and aspartate transaminase (AST) between the male and female NAFLD and control groups (**Table 3**).

**Table 3:** Clinical characteristics of males and females.

	male			female		
	NAFLD(1089)	Control(1004)	P-value	NAFLD(418)	Control(1517)	P-value
ALT(U/L)	28(21,41)	19(15,25)	<0.001	20(16,28)	14(11,18)	<0.001
AST(U/L)	24(20,30)	21(19,25)	<0.001	21(18,26)	19(17,22)	<0.001
TC(mmol/L)	5.19 ± 0.98	4.95 ± 0.94	<0.001	5.27 ± 0.99	5.02 ± 0.89	<0.001
TG(mmol/L)	1.78(1.32,2.50)	1.13(0.82,1.50)	<0.001	1.53(1.12,2.20)	0.89(0.69,1.17)	<0.001
HDL-C(mmol/L)	1.10 ± 0.22	1.26 ± 0.28	<0.001	1.28 ± 0.25	1.51 ± 0.32	<0.001
Hemoglobin(g/L)	158.32 ± 9.59	155.11 ± 9.97	<0.001	134.52 ± 12.57	131.78 ± 11.60	<0.001
Glu(mmol/L)	5.86 ± 1.55	5.37 ± 1.13	<0.001	5.69 ± 1.32	5.11 ± 0.54	<0.001
NC(cm)	38.45 ± 1.97	36.84 ± 1.87	<0.001	35.41 ± 2.39	32.48 ± 2.00	<0.001
Waistline(cm)	96.44 ± 9.16	86.36 ± 7.80	<0.001	90.57 ± 8.58	79.66 ± 7.02	<0.001
AVFA(cm^2^)	106.34 ± 32.47	74.38 ± 24.57	<0.001	125.37 ± 35.47	84.60 ± 29.51	<0.001
Skeletal-muscle(Kg) Homocysteine(umol/L)	39.73 ± 2.85 13.7(11.6,17.2)	42.51 ± 3.02 13.7 (11.6,16.6)	<0.001 0.249	34.35 ± 2.69 9.85(8.50,11.50)	37.05 ± 2.92 9.8(8.4,11.5)	<0.001 0.710

NC, neck circumference; AVFA, abdominal visceral fat area; TC, total cholesterol; TG, triglycerides; HDL-C, high-density lipoprotein cholesterol; Glu, glucose; ALT, alanine aminotransferase; AST, aspartate transaminase.

### 3.4 Combined analysis of homocysteine levels and body composition

**Figure 2:**
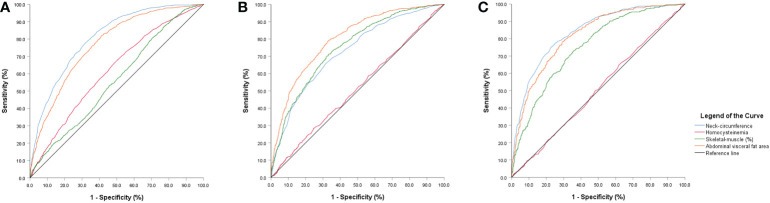
ROC curve analyses **(A)** neck-circumference, abdominal visceral fat area and skeletal-muscle content predictive value for NAFLD in the hyperhomocysteinemia group. **(B)** neck-circumference, abdominal visceral fat area and skeletal-muscle content predictive value for NAFLD in the hyperhomocysteinemia-male group. **(C)** neck-circumference, abdominal visceral fat area and skeletal-muscle content predictive value for NAFLD in the hyperhomocysteinemia-female group.

According to the results of the logistic regression in **Table 4**, we found that skeletal-muscle was associated with a lower risk of NAFLD, whereas BMI, waistline, NC, HHcy were associated with a higher risk of NAFLD. **Figure 2** depicts an ROC curve that included 4028 adults. Area under the curve (AUC) measurements were performed to evaluate the predictive value of anthropometric indices for NAFLD (**Table 5**). As shown in **Table 5**, the AUC is 0.809 in HHcy*NC and HHcy* Skeletal-muscle group, which is the highest value in Total-NAFLD group. The AUC values of HHcy* AVFA, HHcy* Skeletal-muscle, Skeletal-muscle are 0.800, 0.749, 0.748 respectively in Male-NAFLD group. Importantly, p-value of AVFA is 0.881 in **Table 4**. Therefore, HHcy* Skeletal-muscle may have more predictive value for Male-NAFLD. The AUC value of HHcy*NC is 0.837 in Female-NAFLD, which has predictive value for NAFLD. With respect to HHcy, the best NC cut-off point for NAFLD was 37.75 cm (sensitivity 70.1% and specificity 69.8%) in men and 34.45 cm (sensitivity 83.3% and specificity 83.9%) in women. In patients with HHcy, the best AVFA cut-off point for NAFLD was 82.70 cm (sensitivity 82.2% and specificity 66.5%) in men and 106.50 cm (sensitivity 79.2% and specificity 76.3%) in women. Also in patients with HHcy, the best skeletal-muscle content cut-off point for NAFLD was 41.335% (sensitivity 74.2% and specificity 65.6%) in men and 33.88% (sensitivity 62.5% and specificity 82.8%) in women. Based on the above analysis, the best NC cut-off point for NAFLD was 34.45 cm (sensitivity 83.3% and specificity 83.9%) in women with HHcy. and the best skeletal-muscle content cut-off point for NAFLD was 41.335% (sensitivity 74.2% and specificity 65.6%) in men with HHcy.

**Table 4:** Odds ratios and 95% confidence intervals for HHcy, body composition in relation to the risk of NAFLD.

Characteristics	P-value	Odds Ratio (95%Confidence Interval)
Age	<0.001	1.025(1.016–1.034)
Sex		
male		1(ref)
female	<0.001	0.443(0.317-0.619)
BMI(kg/m2)	<0.001	1.367(1.226-1.524)
Waistline(cm)	<0.001	1.206(1.180-1.233)
NC(cm)	0.001	1.305(1.025-1.662)
AVFA(cm2)	0.881	
skeletal-muscle(Kg)	<0.001	0.768(0.704-0.839)
HHcy	0.001	1.026(0.801-1.315)

BMI, body mass index; NC, neck circumference; AVFA, abdominal visceral fat area; These parameters were after adjusting for age, sex, BMI, waistline circumference, HHcy, NC, AVFA, skeletal-muscle.

**Table 5:** Area under the ROC measurements of different anthropometric indices as predictors of NAFLD.

	Characteristics	AUC(95%CI)	P-value
Total-NAFLD	NC (cm)	0.808(0.795,0.821)	<0.001
	HHcy (umol/L)	0.617(0.599,0.635)	<0.001
	Skeletal-muscle (Kg)	0.560(0.542,0.578)	<0.001
	AVFA(cm^2^)	0.770(0.755,0.784)	<0.001
	HHcy*NC	0.809(0.796,0.822)	<0.001
	HHcy* Skeletal-muscle	0.809(0.796,0.822)	<0.001
	HHcy* AVFA	0.780(0.766,0.794)	<0.001
Male-NAFLD	NC(cm)	0.727(0.706,0.749)	<0.001
	Homocysteine(umol/L)	0.515 (0.490,0.539)	0.249
	Skeletal-muscle(Kg)	0.748(0.728,0.769)	<0.001
	AVFA(cm^2^)	0.800(0.781,0.818)	<0.001
	HHcy*NC	0.727(0.706,0.749)	<0.001
	HHcy* Skeletal-muscle	0.749(0.729,0.770)	<0.001
	HHcy* AVFA	0.800(0.781,0.819)	<0.001
Female-NAFLD	NC(cm)	0.836(0.815,0.857)	<0.001
	Homocysteine(umol/L)	0.506 (0.475,0.537)	0.710
	Skeletal-muscle(Kg)	0.751(0.726,0.776)	<0.001
	AVFA(cm^2^)	0.817(0.795,0.839)	<0.001
	HHcy*NC	0.837(0.816,0.858)	<0.001
	HHcy* Skeletal-muscle	0.751(0.726,0.776)	<0.001
	HHcy* AVFA	0.818(0.796,0.839)	<0.001

ROC, receiver operating characteristic; CI, confidence interval; AUC, area under the curve; NC, neck circumference; AVFA, abdominal visceral fat area. The symbol * is a joint effect (as a variable).

## 4 Discussion

Our results suggest that there is a relationship between homocysteine and body composition in the prediction of the probability of NAFLD in healthcare workers. Men were more likely to suffer from NAFLD than women (26). Prior literature has reported that NAFLD more commonly affects men, which may be related to estrogen levels in the body (27). Further, Hcy levels were higher in the NAFLD group than controls, and in the male-NAFLD group than the female-NAFLD group. HHcy alters intracellular lipid metabolism, which may be associated with the pathogenesis NAFLD (28). Finally, skeletal-muscle content was lower in the NAFLD group than controls, which agrees with recent studies that have shown that NAFLD is strongly associated with skeletal-muscle content (29) and that sarcopenia is associated NAFLD severity (30). Waistline, NC, and AVFA measurements were significantly higher in the NAFLD group compared with controls. Increased waist circumference is the main reason for the rapid increase in the prevalence of NAFLD (31). It has also been reported that NC is predictive of NAFLD in an Iranian population. Namely, the best NC cut-off point for NAFLD was 39.25 cm (sensitivity 79% and specificity 69%) in men and 34.85 cm (sensitivity 84% and specificity 64%) in women (32). A cross-sectional study reported that visceral fat area and skeletal-muscle content are predictive of NAFLD (17). One study showed that visceral fat area is associated with NAFLD and related to the degree of advanced fibrosis (33). However, no prior work has taken HHcy into account. Considering the limitations of any of the single indicators noted above, we proposed that HHcy could have a synergistic effect on the development of NAFLD. Patients with NAFLD had higher waistline, NC and AVFA measurements in the HHcy group than the normal-Hcy group, while skeletal-muscle content was lower in the HHcy group than the normal-Hcy group. Although the relevant mechanisms behind this finding are unknown, we hypothesize that insulin resistance plays a key role in this process. First, the literature supports the view that increased serum homocysteine (Hcy) levels may be related to liver fat accumulation in NAFLD (9). Skeletal muscle and the liver are the target organs of insulin. Insulin inhibits the accumulation of fat and leads to the decreased flow of fat into the liver. Insulin resistance can lead to NAFLD and muscle loss (34). In addition, intrahepatic fat added to the accumulation of triglycerides in the liver, insulin resistance could further aggravated (35–37). Importantly, since muscle tissue is key in glucose disposal, sarcopenia itself diminishes insulin-mediated glucose disposal, which is conducive to insulin resistance (38, 39). Second, visceral fat area is an important factor in insulin resistance (40, 41), and was associated with NAFLD. NC is associated with the HOMA-IR index of patients with NAFLD, and insulin resistance may play a key role in disease progression (42). A study in Korea reported that waist circumference is a risk factor for NAFLD and is associated with insulin resistance. The current body of literature therefore indicates that insulin resistance may link HHcy, muscle content, visceral fat area, and waistline. The NC cut-off point for predicting NAFLD was 38 cm in men and 34 cm in women in a Chinese population (18, 42, 43). In our study, the best NC cut-off point for NAFLD was 34.45 cm in women with HHcy, and the best skeletal-muscle content cut-off point for NAFLD was 41.335% in men with HHcy. We therefore believe that including HHcy with body composition analysis may yield a more accurate prediction of NAFLD.

## 5 Limitations

Our results should be considered in the context of several limitations. First, we only speculated that insulin inhibition plays a role in NAFLD, and we will further study this relationship in the future. Second, our study lacks diet-related factors, which will also be studied in a subsequent work. Third, considering the cost and harm of radiation, we used BIA to measure body composition instead of dual energy X-ray absorption. BIA was harmless to the participant’s health, and subjects had better compliance with it. BIA is therefore more conducive to follow-up research. Additionally, there are some limitations of sample representativeness for single center survey, and we will conduct multi-center research in the future.

## 6 Conclusions

Our results indicated that HHcy combined with body composition analysis is predictive of NAFLD. The interaction between skeletal-muscle content, NC, and HHcy may affect the incidence of NAFLD in healthcare workers. Additional attention should therefore be paid to the health of healthcare workers.

## Data availability statement

The original contributions presented in the study are included in the article/supplementary material. Further inquiries can be directed to the corresponding author.

## Ethics statement

The studies involving human participants were reviewed and approved by the Institutional Ethics Committee of Peking University Third Hospital (project: M2021661). The patients/participants provided their written informed consent to participate in this study. Written informed consent was obtained from the individual(s) for the publication of any potentially identifiable images or data included in this article.

## Author contributions

XH, LT, HH, PW: Data curation. XH, and HH: Methodology. XH and PW: Writing – original draft. XH and PW: Writing – review and editing. All authors contributed to the article and approved the submitted version.
